# The Mediating Role of Extra-family Social Relationship Between Personality and Depressive Symptoms Among Chinese Adults

**DOI:** 10.3389/ijph.2022.1604797

**Published:** 2022-09-23

**Authors:** Hanfang Zhao, Hong Shi, Zheng Ren, Minfu He, Xiangrong Li, Yuyu Li, Yajiao Pu, Li Cui, Shixun Wang, Jieyu Zhao, Hongjian Liu, Xiumin Zhang

**Affiliations:** School of Public Health, Jilin University, Changchun, China

**Keywords:** China, adults, depressive symptoms, extra-family social relationship, big-five personality

## Abstract

**Objectives:** This study aims to explore the associations of personality traits and extra-family social relationship with depressive symptoms among Chinese adults.

**Methods:** A nationally representative sample of 29,810 adults aged 16 and above were selected from 2018 CFPS. Personality and depressive symptoms were measured using CBF-PI-15 and the CES-D8 scale. Extra-family social relationship was assessed through the self-rated evaluation. The multiple regression analysis and the PROCESS macro were used for the mediation analysis.

**Results:** Extraversion (OR = 0.807, 95% CI = 0.773, 0.842), agreeableness (OR = 0.795, 95% CI = 0.756, 0.835) and extra-family social relationship (OR = 0.927, 95% CI = 0.913, 0.941) had negative associations with depressive symptoms. Extra-family social relationship could mediate between extraversion and depressive symptoms (Indirect effect = −0.049,95% CI = −0.060, −0.039) as well as agreeableness (Indirect effect = −0.056, 95% CI = −0.068, −0.046) and depressive symptoms. Comparing to females, the indirect effect accounts for a higher proportion of total effect in males.

**Conclusion:** Extra-family social relationship might mediate the association between extraversion and depressive symptoms as well as agreeableness and depressive symptoms.

## Introduction

As a common mental disorder, depression is a leading cause of disability around the world and contributes greatly to the global burden of disease. It has been estimated that a total of 264 million people are influenced by depression in the world [1]. Depression has the features as follows: presenting continuous sadness and lacking enjoyment or interest in the prior enjoyable or rewarding activities. At the same time, it is also able to do harm to appetite and sleep and usually gives rise to tiredness and bad concentration. There can be long or recurrent depression influences, and have a great impact on the functions and rewarding life of people [2]. Depressive symptoms, although not sufficient for clinical diagnosis of depressive disorders, it has been identified as a practical and reliable way for preliminary screening of depression in the general population [3]. In China, depressive symptoms account more for the total personal expected medical spending than depression [4]. The associations between depressive symptoms and demographic characteristics, physiological health, behavioral factors and social capital among Chinese people have been investigated [5]. Notably, the associations of personality traits and depressive symptoms have received more and more attention abroad in recent years [6–8]. Given that previous Chinese studies on the association of personality traits and depressive symptoms are based on specific age ranges, limited sample regions (data often gathered in a few cities or provinces), and small or medium sample size (less than 10,000 respondents) [9, 10], there is a great need for more comprehensive studies based on nationally representative data.

For the purpose of explaining the personality-depression association, at least 6 alternative and partially overlapping theoretical models, including the common cause, spectrum, vulnerability, precursor, pathoplasty, and scar/complication, have already been put forward [11–15]. To be specific, the personality traits strongly associated with depressive symptoms lie in neuroticism (N), extraversion (E) and conscientiousness (C) in both healthy and patient populations in accordance with the published reviews [16–18]. Moreover, it is defined that high neuroticism, low extraversion, and low conscientiousness served as the vulnerable type for depressive symptoms [19]. However, in a meta-analysis, Malouff et al. (2005) showed that low agreeableness (A) is another personality trait associated with mental disorders [20]. The researches on the association of agreeableness, openness (O) and depressive symptoms has not reached a consistent conclusion. In a meta-analysis of 175 studies, it was found that agreeableness and openness were largely unrelated to depressive symptoms [16], while some studies indicated that agreeableness and openness are negatively associated with depressive symptoms [21, 22]. The different research results may be attributed to the differences in research samples, personality traits and depressive symptoms measurement, national cultural background as well as adjustment for potential confounding factors.

Notably, a meta-analysis of 10 cohort studies indicated that heterogeneity observed in the associations between personality and depressive symptoms may be related to social factors those unmeasured or measured imprecisely in the previous studies [17]. Compared with intra-family settings, individuals in extra-family settings (e.g., work, communities) are more likely to experience interpersonal distress. It is thought that the relationship between personality and depressive symptoms is rooted in a fundamental motivational system. The theory of Dynamics of Personality States points out that personality states may be elicited by different situations, options for such situational variables could be social roles (e.g., mother, CEO) or cultural cues [23]. Personality states not only serve as the counterparts of personality traits but also represent behavioral syndromes that indicate what the person on the whole is doing [24]. Personality states and personality traits can be measured with the same scale, and the personality scales about social interaction more largely reflects the personality state when socializing outside the family. Compared with people who score lower on extraversion, people scoring higher on extraversion go through more positive emotions, have a tendency to be friendly, cheerful and sociably, and are active as well as pursue social interaction [25]. Compared with people who score lower on agreeableness, people scoring higher on agreeableness show larger trust, altruism, straightforwardness, compliance, modesty, trust and tender-mindedness [25]. As a result, it is possible that personality states when individuals play roles in extra-family associated with extra-family social relationship.

The association between depression and social relationships has long been of interest. The theory of social determination proposes that individuals have the need for relatedness, that is, having close and meaningful relationships with others. If the need for a relationship is not sufficiently met, this may lead to loneliness and depression [26]. In a large nationally representative community cohort study, it has found that after accounting for baseline depression and other important potential confounders, the quality of social relationship predicted future depression [27], and other studies also indicated the negative relationship between social relationship and depression [28]. Conceptually, social relationship is likely to have an impact on mental health outcomes through multiple mechanisms including influence on health-related behaviors, engagement in social activities, transfer and exchange of social support, and access to material resources [29]. Empirically, social isolation and negative social interactions are associated with depression [30]. Social relationship includes intra-family and extra-family social relationship. However, there is no definitive conclusion on whether extra-family social relationship is associated with depressive symptoms. In previous studies, the conclusion of whether social relationships among friends, as well as in work settings, were associated with depressive symptoms, was not consistent [28]. Therefore, it is meaningful to explore the correlation between extra-family social relationship and depressive symptoms.

In the common cause model, it is posited that personality and depression are from common determinants, including genetic factors, explaining the relationship [14]. Gender serves as the most apparent genetic factor, with significant differences in physiology and social roles between men and women. Due to physiological cycle and other reasons, women’s hormones change more frequently, and they are almost twice as likely as men to have depressive symptoms throughout their life cycle [31]. However, researches on the mechanism of gender differences in depression is not comprehensive, and most of the researches focuses on financial comfort, social support, physiological health and gender equity [31, 32]. And there is still lack of research about whether the associations between personality traits and depression varies in different gender groups.

The 2018 China Family Panel Studies (CFPS) was the first time that personality traits had been included in a large-scale national survey in China. The study used the national representative sample data to analyze the association of personality and depressive symptoms among Chinese adults. Considering the poor variability of personality traits, this study introduced extra-family social relationship that is not only closely related to daily life among adults but also can be promoted through some strategies and measures. This study examined whether extra-family social relationship mediated between personality traits and depressive symptoms, which is lacking in existing studies. Moreover, there was also an examination of whether gender difference exists in the relationship of personality and extra-family social relationship with depressive symptoms.

Based on the above studies on depressive symptoms, personality traits, and extra-family social relationship, we hypothesized that: (1) path c: personality traits (extraversion and agreeableness) are related to depressive symptoms. (2) path a & b: the association between personality traits (extraversion and agreeableness) and depressive symptoms among Chinese adults is mediated by extra-family social relationship.

## Methods

### Data Sources and Sample Composition

The data were provided by the 2018 CFPS personal database, which is one of the national representatives for annual longitudinal review and approval by the Institute of Social Science Survey (ISSS) of Peking University [33]. All respondents were required to submit the written informed consent before the completion of the investigation. Using the method of ‘multistage probability proportional-to-size sampling’, the CFPS investigated about 15,000 households nationwide and interviewed all family members of each sampled household. A total of 30,105 participants aged 16 or above completed the full version with 8 questions of the Center for Epidemiologic Studies Depression (CES-D8) questionnaire. There were 29,810 subjects included in the final analytical sample after deleting the variables of extraversion, agreeableness, and extra-family social relationship containing missing values, as well as completing the covariate variables containing missing values.

### Measures

#### Personality Traits

Personality traits were assessed using the 15-item short version of the Big Five Inventory (CBF-PI-15). This is a brief self-report based on the five-factor model that is used to survey the personality traits of Chinese people [34]. Extraversion and agreeableness are two of the five dimensions. Each dimension includes 3 items. Participants responded on the 5-point scale ranging from 1 (strongly disagree) to 5 (strongly agree). After correcting the reversely scored items (one for each dimension) and averaging the score of each dimension, the final score of each personality was calculated, with higher scores indicating higher levels of the corresponding personality trait [35]. Previous research using the CBF-PI-15 has shown that it demonstrates excellent reliability and validity across diverse samples [34, 36, 37].

#### Extra-Family Social Relationship

Extra-family social relationships were measured by asking respondents the following question: “How would you rate your extra-family relationship?”, and the answer ranged from 0 (worst) to 10 (best). Higher scores indicate better extra-family social relationship.

#### Depressive Symptoms

Depressive symptoms of the participants in this study were measured using the 8-item Center for Epidemiological Studies Depression (CES-D8) scale. It measures the frequency of symptoms over the last week and rates on a 4-point scale: 0 for “less than a day,” 1 for “1–2 days,” 2 for “3–4 days” and 3 for “5–7 days” [38]. 2 of the 8 items are scored reversely. A total score of CES-D8 was calculated after correcting the reversely scored items and summing the scores, and higher scores suggested a greater likelihood of depression. It has been shown that CES-D8 with high reliability is an effective short form of CES-D as the original longer form [39]. The CES-D8 score varies from 0 to 24, and a score ≥ 9 is used to identify people with clinically significant depressive symptoms [40].

#### Covariate Assessment

Demographic characteristics included gender, age, marital status, place of residence, and educational level. Age was divided into 16∼44, 45∼59, and 60∼. Marital status consisted of never married, married or cohabitating, and widowed or divorced. Place of residence included rural and urban areas. Educational level was classified according to China’s education system setting: 1) primary school or below; 2) middle school; 3) high school; 4) college degree or above.

This study considered two physiological health factors: BMI and self-rated health. BMI was calculated by dividing body weight by the square of body height (kg/m). In addition, within the revised Asia-Pacific BMI criteria by the World Health Organization [41], it is pointed out that BMI < 18.5, 18.5 ≤ BMI < 24.0, 24.0 ≤ BMI < 28.0, BMI ≥ 28.0 were defined as underweight, normal, overweight and obesity, respectively. Self-rated health was measured by asking respondents how they evaluated their health status, which was categorized into good, fair and bad.

Social status and self-rated income were assessed by 5-point scale questions, higher score indicated higher level of social status and self-rated income in the local area of the participant. Social status was evaluated by requiring respondents to rate their own social status in the local area, which was categorized into high, medium, and low. Self-rated income was assessed through self-rated income level in the local area. Since some of the participants were students, self-rated income was divided into medium or low, high and inapplicable.

Physical activities were measured by asking respondents about the frequency of physical exercise in the last week. These responses were grouped into never (0), sometimes (1∼3) and often (≥4). Drinking status was determined by asking whether they had drunk alcohol more than three times a week in the past month (yes or no). Smoking status was determined by asking whether they had smoked in the past month (yes or no).

Family relationship was included as an adjustment variable since this study focused on the extra-family social relationship. It was assessed by self-rated relationship with spouses, parents, and children. The participants were asked to score their relationship with their spouses, parents, and children from 1 (lowest) to 5 (highest), respectively, in which a higher score indicated a higher level of social status. The average score of the above family members was taken as the final score of the family relationship.

### Statistical Analyses

Data were managed and analyzed using the SPSS software version 24 (IBM, Armonk, NY, United States). First of all, the *t*-test, analysis of variance (ANOVA) and linear regression analysis were used to assess the differences of personality scores in basic characteristics. Secondly, the associations of personality traits, extra-family social relationship, and depressive symptoms were analyzed using binary logistic regression models. The odds ratios (ORs) and 95% confidence intervals (CIs) were adjusted for potential confounding factors. Thirdly, Pearson correlations were conducted to investigate the relationship of personality traits, extra-family social relationship, and depressive symptoms. Finally, the mediating role of extra-family social relationship in personality traits and depressive symptoms was tested and reconfirmed using the multiple linear regression analysis and the PROCESS macro. Based on 5,000 bootstrapped samples, the 95% bootstrap CI was calculated and *p* value < 0.05 was considered significant.

## Results

### Sample Characteristics and Personality Traits

The results showed that the prevalence of depressive symptoms among Chinese adults was 21.3% (6,348/29,810). Of all participants, 50.4% were females, and the number of participants aged 16∼44, 45∼59 and 60∼ were 12,984 (43.6%), 9,018 (30.3%) and 7,808 (26.1%), respectively.

All characteristics were associated with extraversion and all characteristics except BMI were associated with agreeableness (*p* < 0.05). Table 1 describes the sample characteristics of participants with different scores of extraversion and agreeableness in detail.

**TABLE 1:** Sample characteristics of the participants and the distributions of extraversion and agreeableness in categorical items (N = 29,810). (China, 2018).

	Variables	Sample (*n* = 29,810)	Extraversion		Agreeableness	
			Mean (SD)	*p*	Mean (SD)	*p*
Gender	Male	14,790 (49.6)	3.36 (0.71)	<0.001	3.79 (0.61)	<0.001
	Female	15,020 (50.4)	3.32 (0.70)		3.85 (0.61)	
Age (year)	16∼44	12,984 (43.6)	3.29 (0.69)	<0.001	3.77 (0.59)	<0.001
	45∼59	9,018 (30.3)	3.34 (0.71)		3.84 (0.60)	
	60∼	7,808 (26.1)	3.43 (0.72)		3.89 (0.62)	
Area of residence	Rural	14,682 (49.3)	3.35 (0.70)	0.011	3.80 (0.61)	<0.001
	Urban	15,128 (50.7)	3.33 (0.71)		3.84 (0.60)	
Marital status	Never married	4,744 (15.9)	3.32 (0.71)	0.007	3.76 (0.59)	<0.001
	Married or cohabitating	23,132 (77.6)	3.34 (0.70)		3.83 (0.61)	
	Widowed or divorced	1934 (6.5)	3.38 (0.74)		3.90 (0.63)	
Educational level	Primary school or below	12,641 (42.4)	3.37 (0.71)	<0.001	3.82 (0.63)	0.001
	Middle school	8,869 (29.8)	3.34 (0.70)		3.81 (0.60)	
	High school	4,766 (16.0)	3.32 (0.71)		3.83 (0.58)	
	College degree or above	3,534 (11.8)	3.28 (0.70)		3.85 (0.58)	
BMI	Underweight	13,921 (46.7)	3.31 (0.70)	<0.001	3.82 (0.61)	0.134
	Normal	14,144 (47.4)	3.37 (0.70)		3.82 (0.61)	
	Overweight	1,567 (5.3)	3.38 (0.74)		3.80 (0.61)	
	Obesity	178 (0.6)	3.46 (0.70)		3.75 (0.58)	
Self-rated health	Fair or bad	21,034 (70.6)	3.36 (0.70)	<0.001	3.84 (0.59)	<0.001
	Good	8,776 (29.4)	3.30 (0.72)		3.79 (0.64)	
Social status	Medium or low	20,941 (70.2)	3.29 (0.71)	<0.001	3.79 (0.60)	<0.001
	High	8,869 (29.8)	3.47 (0.60)		3.90 (0.61)	
Self-rated income	Medium or low	21,553 (72.3)	3.31 (0.71)	<0.001	3.82 (0.60)	<0.001
	High	6,410 (21.5)	3.44 (0.68)		3.86 (0.61)	
	Inapplicable	1847 (6.2)	3.35 (0.71)		3.77 (0.60)	
Exercise	Never	15,247 (51.2)	3.29 (0.71)	<0.001	3.78 (0.61)	<0.001
	Sometimes	4,748 (15.9)	3.31 (0.69)		3.82 (0.58)	
	Often	9,815 (32.9)	3.44 (0.70)		3.89 (0.61)	
Smoking	No	21,199 (71.1)	3.32 (0.70)	<0.001	3.83 (0.60)	<0.001
	Yes	8,611 (28.9)	3.40 (0.71)		3.79 (0.62)	
Drinking	No	25,320 (84.9)	3.33 (0.70)	<0.001	3.83 (0.61)	<0.001
	Yes	4,490 (15.1)	3.43 (0.71)		3.78 (0.61)	
Family relationship		4.38 ± 0.68	—	<0.001	—	<0.001

Note: Differences were assessed using linear regression for family relationship, *t* test for gender, area of residence, self-rated health, social status, smoking and drinking, and analysis of variance (ANOVA) for other variables.

### Personality Traits, Extra-family Social Relationship, and Depressive Symptoms

A higher level of extraversion (OR = 0.757, 95% CI = 0.728, 0.788), agreeableness (OR = 0.733, 95% CI = 0.701, 0.767) and extra-family social relationship (OR = 0.885, 95% CI = 0.873, 0.898) associated with a lower level of depressive symptoms among Chinese adults. After adjusting for potential confounding factors, the OR value changed to 0.807 (95% CI = 0.773,0.842), 0.795 (95% CI = 0.756,0.835) and 0.927 (95% CI = 0.913,0.941), respectively. Whetherbefore or after adjustment, extraversion, agreeableness and extra-family social relationship had a significant association with depressive symptoms. Data were shown in Table 2.

**TABLE 2:** Extraversion, agreeableness and extra-family social relationship as predictors for depressive symptoms. (China, 2018).

Variables	Unadjusted OR (95% CI)	*p*	Adjusted OR (95%CI)	*p*
Extraversion	0.757 (0.728, 0.788)	<0.001	0.807 (0.773, 0.842)^a^	<0.001
Agreeableness	0.733 (0.701, 0.767)	<0.001	0.795 (0.756, 0.835)^b^	<0.001
Extra-family social relationship	0.885 (0.873, 0.898)	<0.001	0.927 (0.913, 0.941)^c^	<0.001

a Adjusted for all covariates.

b Adjusted for all covariates except body mass index (BMI).

c Adjusted for all covariates.

Note: No depressive symptoms (Score of 8-item Center for Epidemiological Studies Depression < 9) is the reference category for the outcome variable. OR represents the odds ratio; 95% CI represents 95% confidence intervals; *P* was calculated by analysis of binary logistic regression.

### Correlation Analysis

The Pearson correlations (two-tailed) of extraversion, agreeableness, extra-family social relationship, and depressive symptoms were presented in Supplementary Table S1. It was found that all correlation coefficients were significant (*p* < 0.001). Extraversion, agreeableness, and extra-family social relationship were negatively correlated with depressive symptoms.

### Testing for the Mediation Effect

The multiple linear regression analysis was adopted to test whether extra-family social relationship could mediate the association between extraversion and depressive symptoms as well as agreeableness and depressive symptoms. The confounding factors were controlled in the analysis of the mediation effect. Table 3 displays the testing results. Firstly, extraversion and agreeableness had a significant association with depressive symptoms (Estimate = −0.477, 95% CI = −0.538, −0.416, *p* < 0.001 for extraversion; estimate = −0.552, 95% CI = −0.623, −0.481, *p* < 0.001 for agreeableness). Secondly, extraversion and agreeableness had a significant association with extra-family social relationship (Estimate = 0.349, 95% CI = 0.318, 0.380, *p* < 0.001 for extraversion; estimate = 0.398, 95% CI = 0.363, 0.434, *p* < 0.001 for agreeableness). Thirdly, extraversion and agreeableness were both controlled, and extra-famly social relationship had an association with depressive symptoms (Estimate = −0.140, 95% CI = −0.162, −0.117, *p* < 0.001 for extraversion; estimate = −0.141, 95% CI = −0.164, −0.119, *p* < 0.001 for agreeableness). In addition, the mediation effects were tested in males and females, respectively, which presented in Supplementary Tables S2, S3. Similar to the overall participants, pathways remained statistically significant in both male and female groups (*p* < 0.001).

**TABLE 3:** Testing the mediation effect of extra-family social relationship in the association between extraversion, agreeableness and depressive symptoms (*n* = 29,810). (China, 2018).

Variables	Estimate	95% CI	*t*	*R* ^*2*^	*F*
Extraversion → Extra-family social relationship → Depressive symptoms^a^					
Step 1: Extraversion predicts depressive symptoms					
Independent variable: extraversion	−0.477	(−0.538, −0.416)	−15.397***	0.153	376.488***
Dependent variable: depressive symptoms					
Step 2: Extraversion predicts extra-family social relationship					
Independent variable: extraversion	0.349	(0.318, 0.380)	22.274***	0.098	226.896***
Dependent variable: extra-family social relationship					
Step 3:Extra-family social relationship predicts depressive symptoms					
Independent variable: extraversion	−0.428	(−0.489, −0.367)	−13.741***	0.157	362.897***
Mediator: extra-family social relationship	−0.140	(−0.162, −0.117)	−12.097***		
Dependent variable: depressive symptoms					
Agreeableness → Extra-family social relationship → Depressive symptoms^b^					
Step 1: Agreeableness predicts depressive symptoms					
Independent variable: agreeableness	−0.552	(−0.623, −0.481)	−15.265***	0.151	398.725***
Dependent variable: depressive symptoms					
Step 2: Agreeableness predicts extra-family social relationship					
Independent variable: agreeableness	0.398	(0.363, 0.434)	21.810***	0.098	242.321***
Dependent variable: extra-family social relationship					
Step 3: Extra-family social relationship predicts depressive symptoms					
Independent variable: agreeableness	−0.496	(−0.567, −0.424)	−13.631***	0.155	382.815***
Mediator: extra-family social relationship	−0.141	(-0.164, -0.119)	−12.229***		
Dependent variable: depressive symptoms					

a Adjusted for all covariates.

b Adjusted for all covariates except body mass index (BMI).

Values were calculated by multiple linear regression analysis.

***p < 0.001.

### Reconfirming for the Mediation Effect

**FIGURE 1:**
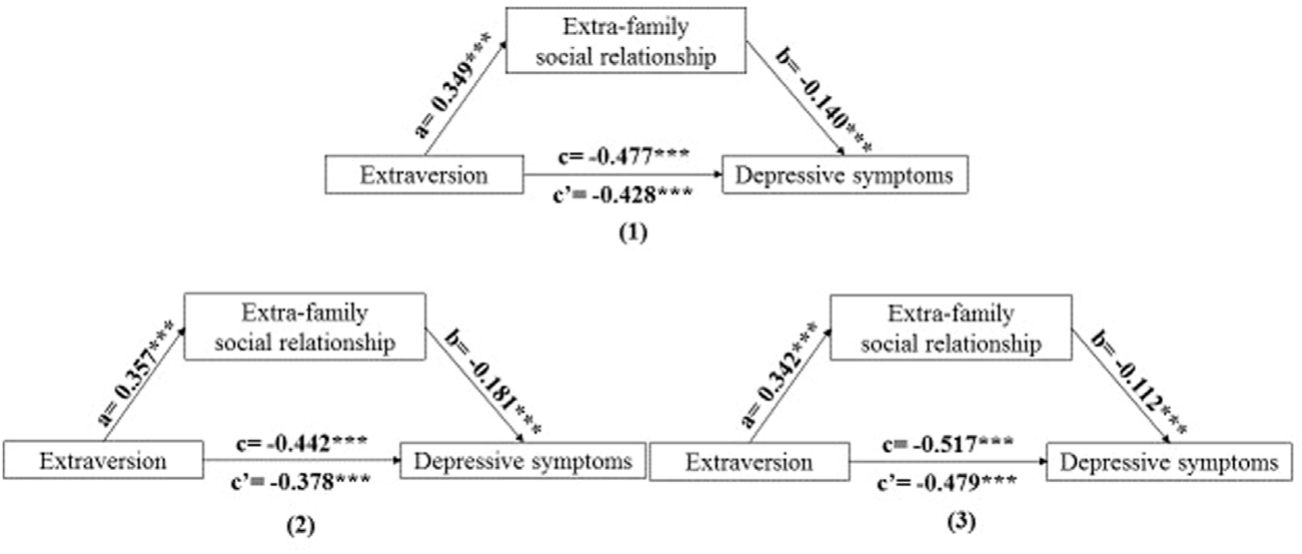
The mediating role of extra-family social relationship on the association between extraversion and depressive symptoms. Note: (1) overall; (2) males; (3) females. a path: a significant association between extraversion and extra-family social relationship. b path: a significant association between extra-family social relationship and depressive symptoms after controlling extraversion. c’ path: a significant association between extraversion and depressive symptoms after controlling extra-family social relationship. c = c’+ab. ***p < 0.001. (China, 2018).

**FIGURE 2:**
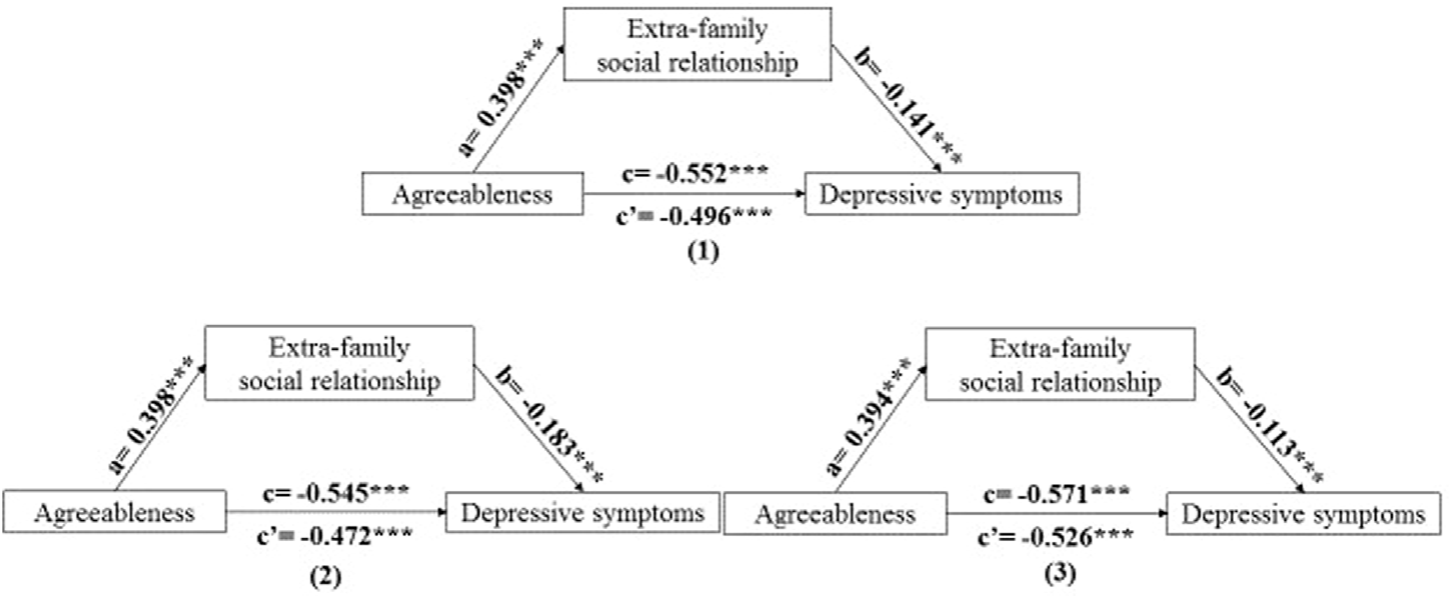
The mediating role of extra-family social relationship on the association between agreeableness and depressive symptoms. Note: (1) overall; (2) males; (3) females. a path: a significant association between agreeableness and extra-family social relationship. b path: a significant association between extra-family social relationship and depressive symptoms after controlling agreeableness. c’ path: a significant association between agreeableness and depressive symptoms after controlling extra-family social relationship. c = c’+ab. ***p < 0.001. (China, 2018).

The effect was reconfirmed through the PROCESS macro (Model 4), which showed a reduced direct effect of extraversion and agreeableness on depressive symptoms, respectively. It was found that extraversion (Indirect effect = −0.049, 95% CI = −0.060, −0.039) and agreeableness (Indirect effect = −0.056, 95% CI = −0.068, −0.046) had indirect effects on depressive symptoms through extra-family social relationship among overall participants. Moreover, it was also found that extraversion and agreeableness had indirect effects on depressive symptoms through extra-family social relationship among males (Indirect effect = −0.064, 95% CI = −0.080, −0.051 for extraversion; indirect effect = −0.073, 95% CI = −0.090, −0.057 for agreeableness) and females (Indirect effect = −0.038, 95% CI = −0.052, −0.026 for extraversion; indirect effect = −0.045, 95% CI = −0.061, −0.030 for agreeableness), respectively. Data were shown in Table 4. Thus, it can be concluded that extra-family social relationship can partially mediate the association between extraversion and depressive symptoms as well as agreeablenessand depressive symptoms. The mediation models were illustrated in Figures 1, 2.

**TABLE 4:** Direct and indirect effects of personality traits on depressive symptoms. (China, 2018).

Path	Effect	95% CI	Accounting for total effect, %
Overall			
Extraversion → depressive symptoms^a^	−0.428	−0.489, −0.367	89.7
Extraversion → Extra-family social relationship → Depressive symptoms^a^	0.349×(−0.140) = −0.049	−0.060, −0.039	10.3
Total effect^a^	−0.477	−0.538, -0.416	—
Agreeableness → depressive symptoms^b^	−0.496	−0.567, −0.424	89.9
Agreeableness → Extra-family social relationship → Depressive symptoms^b^	0.398×(−0.141) = −0.056	−0.068, −0.046	10.1
Total effect^b^	−0.552	−0.623, −0.481	—
Males			
Extraversion → depressive symptoms^a^	−0.378	−0.461, −0.294	85.5
Extraversion → Extra-family social relationship → Depressive symptoms^a^	0.357×(−0.181) = −0.064	−0.080, -0.051	14.5
Total effect^a^	−0.442	−0.526, −0.359	—
Agreeableness → depressive symptoms^b^	−0.472	−0.570, −0.374	86.6
Agreeableness → Extra-family social relationship → Depressive symptoms^b^	0.398×(−0.183) = −0.073	−0.090, −0.057	13.4
Total effect^b^	−0.545	−0.643, −0.447	—
Females			
Extraversion → depressive symptoms^a^	−0.479	−0.568, −0.390	92.6
Extraversion → Extra-family social relationship → Depressive symptoms^a^	0.342×(−0.112) = −0.038	−0.052, −0.026	7.4
Total effect^a^	−0.517	−0.606, −0.429	—
Agreeableness → depressive symptoms^b^	−0.526	−0.629, −0.423	92.1
Agreeableness → Extra-family social relationship → Depressive symptoms^b^	0.394×(−0.113) = −0.045	−0.061, −0.030	7.9
Total effect^b^	−0.571	−0.673, −0.468	—

a Adjusted for all covariates.

b Adjusted for all covariates except body mass index (BMI).

Values were calculated by the PROCESS macro.

## Discussion

There are three major findings in this study. (1) Lower levels of extraversion and agreeableness were related to higher level of depressive symptoms among Chinese adults. (2) Poor extra-social relationship was related to higher level of depressive symptoms. (3) Extra-family social relationship mediated the association between extraversion and depressive symptoms as well as agreeableness and depressive symptoms. These findings suggested that extraversion, agreeableness and extra-family social relationship exerted a certain function on depressive symptoms in Chinese adults, which should be an important concern when formulating intervention strategies for preventing and treating depressive symptoms.

This study found that Chinese adults with higher levels of extraversion and agreeableness were less likely to have depressive symptoms, which was supported by previous studies in Chinese specific populations (such as the unemployed and college students) [9, 10, 42]. Extraversion includes sociability, activity, assertiveness, and positive emotionality [43], which is believed to be rooted in a fundamental motivational system (such as behavioral activation system) [44]. Extraverted individuals behave more frequently in a style that inspires pleasant feelings, expresses more positive ideas, and can be more likely to encounter objectively pleasant events, compared with their introverted counterparts [23]. Positive thoughts and pleasant feelings may protect extraverted individuals from the onset of depressive symptoms. Agreeableness includes altruism, tender-mindedness, trust, and modesty [43], which is often described as emotional intelligence [45]. Agreeable individuals are more inclined to consider others, listen to others, comfort and encourage others when they are frustrated. Conversely, when they encounter negative life events, they are also more likely to receive assistance and encouragement from others, overcome difficulties, and stay away from negative emotions and depressive symptoms.

Previous studies on the association between extra-family social relationship and depressive symptoms provided conflicting results [28]. This study showed that better extra-family social relationship may be related to higher level of depressive symptoms among Chinese adults. It was confirmed that the association between social relationship and depression were attributed to the effects of perceived emotional support, perceived instrumental support, and large and diverse social networks on depression. A community cohort study has reported that social network is an important protective factor against depression [46]. And it has been reported that individuals in family-dependent or private restricted support networks have the highest risk of depression and other mental health conditions. In contrast, those in a locally integrated network have a lower risk of adverse mental health outcomes [47]. The particularly high prevalence of private restricted social networks in urban China may be associated with rapid urbanization over the last 20 years, which is associated with a decline in social ties and a change in the structure of personal networks, especially for migrants [48]. What’s more, in urban China (in common with other sites), a high proportion residents live with their spouse only, while not with their children or parents, whose social relationships with friends and neighbours were more important to protect themselves from depressive symptoms [49]. Moreover, it has been showed that distrustful, disrespectful and uncooperative social relationships at work are independent predictors of physician-diagnosed depression and antidepressant treatment [50]. In addition, it has also been supported that subjective components of social relationships are more critical to one’s health than objective characteristics of one’s social network [27]. In some areas, coworkers and friends could better understand one’s work and life in the confusion, frustration and bottlenecks, and be more likely to offer targeted recommendations and substantive help. With a high level of emotional support and structural support, individuals can not only get informational and tangible help or a solution way, but also pour negative emotions out, obtain sympathy, belongingness, esteem, comfort and useful advice during times of crisis or hardship [51]. Finally, they are more likely to overcome difficulties, get out of negative emotions, and stay away from depressive symptoms.

It was also found that higher levels of extraversion and agreeableness may be related to better extra-family social relationship, which in turn are related to higher level of depressive symptoms. People who score higher on extraversion have tendencies to facilitate better coping with stress. Better dealing with stress may result in a lower need for social support [52], which can increase the perceived availability of social support. In the meantime, extraversion are supposed to be associated with preferences to pursue social interactions whenever are in face of the stress. The individuals who tend to be agreeable are likely to better set up a wider social support network, which may provide the basis for seeking support in face of different stressors [53]. Generally, extraverted and agreeable ones at work, were more likely to establish trustful, respectful and cooperative social relationships with coworkers, bosses and supervisors. When getting along with friends, they are more friendly, positive and optimistic, more empathetic and easier to establish close and long-term relationship with friends. In contrast, introverted and/or disagreeable individuals are often unable to establish lasting and close social relationships with friends and coworkers. According to the theory of social determination [26], having positive social relationships is of great importance to individuals. Close relationships with coworkers, neighbors, and communities have significantly lower rates of depressive symptoms than private restricted networks [46]. In addition, establishing a meaningful relationship with others is the main foundation for individuals to get the emotional outlet and social support, reduce loneliness and integrate into the group [54, 55]. As a result, it reduces the risk of depressive symptoms.

Extra-family social relationship played a stronger mediating role in males, which may be related to Chinese cultural background. Nowadays, although women have the freedom to work in China, it is still common for many families that men work to earn money and women focus on their families. In a Chinese study of marriage satisfaction and gender norms, it is pointed out that husbands may have specific self-images of traditional gender division norms by examining differences between husbands and wives in housework and economic contributions [56]. Generally, compared with women, men’s relationships with colleagues and friends may be more important in their lives. In addition, a study on the relationship between social skills and happiness indicated that men scored higher in all social skills-related factors. The factors such as self-expression in social settings and the ability to say no and cut off social interactions have direct and significant effects on happiness among men [57]. In other words, men are more capable than women of achieving extra-family social relationship. Generally, men with higher level of extraversion and agreeableness may build good extra-family social relationship [25], which is likely to provide good social support for men when they encounter negative events in their lives, including emotional and instrumental support [29], helping them overcome difficulties and reduce the risk of depressive symptoms.

In the context of industrialization, urbanization, and an aging population, *The Outline of the Healthy China 2030 Plan* proposed that it is a necessity to advocate the people-oriented development concept and promote the people’s all-around health. Considering the mediating role of extra-family social relationship between personality traits and depressive symptoms, community managers, and school/work leaders should make joint efforts to improve introverted and disagreeable individuals’ extra-family social relationships, such as strengthening mental health education, establishing mutual help groups, increasing community activities and league construction in workplace, setting up psychological counseling rooms and other strategies.

Major limitations of this study include the nature of the cross-sectional study, which may make it difficult to correlate causes with effects from the findings. In addition, the data were collected through self-reporting, which could yield recall biases as well.

### Conclusion

This study demonstrates the necessity for paying attention to the relationship of personality traits and depressive symptoms among Chinese adults. Lower extraversion, agreeableness, and poor extra-family social relationship could be related to higher level of depressive symptoms. Furthermore, extra-family social relationship might mediate the association between extraversion and depressive symptoms as well as agreeableness, and depressive symptoms.
