# Supplementary material for: The Mediating Role of Extra-family Social Relationship Between Personality and Depressive Symptoms Among Chinese Adults
Source: Int J Public Health. 2022 Sep 23;67:1604797. doi: 10.3389/ijph.2022.1604797 (PMC9537382; doi:10.3389/ijph.2022.1604797)
Supplement: Supplementary file 1 [file DataSheet1.docx]

**Supplementary Table S1.** Correlations of extraversion, agreeableness, extra-family social relationship and depressive symptoms. (China, 2018)

| **Variables** | **1** | **2** | **3** | **4** |
| --- | --- | --- | --- | --- |
| 1. Extraversion | 1 |  |  |  |
| 2. Agreeableness | 0.067^***^ | 1 |  |  |
| 3. Extra-family social relationship | 0.167^***^ | 0.165^***^ | 1 |  |
| 4. Depressive symptoms | -0.122^***^ | -0.199^***^ | -0.137^***^ | 1 |

Note: 8-item Center for Epidemiological Studies Depression scores of nine and above indicates tendency of depressive symptoms.

^***^ *P* < 0.001.

**Supplementary Table S2.** Testing the mediation effect of extra-family social relationship in the association between extraversion, agreeableness and depressive symptoms in males (n = 29810). (China, 2018)

| **Variables** | **Estimate** | **95%CI** | ***t*** | ***R^2^*** | ***F*** |
| --- | --- | --- | --- | --- | --- |
| **Extraversion → Extra-family social relationship → Depressive symptoms ^a^** |  |  |  |  |  |
| Step 1: Extraversion predicts depressive symptoms |  |  |  |  |  |
| Independent variable: extraversion | -0.442 | (-0.525, -0.359) | -10.436^***^ | 0.131 | 168.327^***^ |
| Dependent variable: depressive symptoms |  |  |  |  |  |
| Step 2: Extraversion predicts extra-family social relationship |  |  |  |  |  |
| Independent variable: extraversion | 0.357 | (0.315, 0.400) | 16.442^***^ | 0.102 | 125.885^***^ |
| Dependent variable: extra-family social relationship |  |  |  |  |  |
| Step 3:Extra-family social relationship predicts depressive symptoms |  |  |  |  |  |
| Independent variable: extraversion | -0.378 | (-0.461, -0.294) | -8.866^***^ | 0.139 | 166.626^***^ |
| Mediator: extra-family social relationship | -0.181 | (-0.213, -0.149) | -11.209^***^ |  |  |
| Dependent variable: depressive symptoms |  |  |  |  |  |
| **Agreeableness → Extra-family social relationship → Depressive symptoms ^b^** |  |  |  |  |  |
| Step 1: Agreeableness predicts depressive symptoms |  |  |  |  |  |
| Independent variable: agreeableness | -0.545 | (-0.643, -0.447) | -10.947^***^ | 0.129 | 178.465 |
| Dependent variable: depressive symptoms |  |  |  |  |  |
| Step 2: Agreeableness predicts extra-family social relationship |  |  |  |  |  |
| Independent variable: agreeableness | 0.398 | (0.348, 0.448) | 15.598^***^ | 0.100 | 133.523^***^ |
| Dependent variable: extra-family social relationship |  |  |  |  |  |
| Step 3: Extra-family social relationship predicts depressive symptoms |  |  |  |  |  |
| Independent variable: agreeableness | -0.472 | (-0.570, -0.374) | -9.448^***^ | 0.137 | 176.027^***^ |
| Mediator: extra-family social relationship | -0.183 | (-0.214, -0.151) | -11.312^***^ |  |  |
| Dependent variable: depressive symptoms |  |  |  |  |  |

Note: ^a^ Adjusted for all covariates.

^b^ Adjusted for all covariates except body mass index (BMI).

^***^ *P* < 0.001

**Supplementary Table S3.** Testing the mediation effect of extra-family social relationship in the association between extraversion, agreeableness and depressive symptoms in females (n = 29810). (China, 2018)

| **Variables** | **Estimate** | **95%CI** | ***t*** | ***R^2^*** | ***F*** |
| --- | --- | --- | --- | --- | --- |
| **Extraversion → Extra-family social relationship → Depressive symptoms ^a^** |  |  |  |  |  |
| Step 1: Extraversion predicts depressive symptoms |  |  |  |  |  |
| Independent variable: extraversion | -0.517 | (-0.606, -0.429) | -11.477^***^ | 0.158 | 211.600^***^ |
| Dependent variable: depressive symptoms |  |  |  |  |  |
| Step 2: Extraversion predicts extra-family social relationship |  |  |  |  |  |
| Independent variable: extraversion | 0.342 | (0.297, 0.386) | 15.149^***^ | 0.100 | 124.950^***^ |
| Dependent variable: extra-family social relationship |  |  |  |  |  |
| Step 3:Extra-family social relationship predicts depressive symptoms |  |  |  |  |  |
| Independent variable: extraversion | -0.479 | (-0.568, -0.390) | -10.563^***^ | 0.161 | 200.381^***^ |
| Mediator: extra-family social relationship | -0.112 | (-0.144, -0.080) | -6.788^***^ |  |  |
| Dependent variable: depressive symptoms |  |  |  |  |  |
| **Agreeableness → Extra-family social relationship → Depressive symptoms ^b^** |  |  |  |  |  |
| Step 1: Agreeableness predicts depressive symptoms |  |  |  |  |  |
| Independent variable: agreeableness | -0.571 | (-0.673, -0.468) | -10.909^***^ | 0.155 | 224.102^***^ |
| Dependent variable: depressive symptoms |  |  |  |  |  |
| Step 2: Agreeableness predicts extra-family social relationship |  |  |  |  |  |
| Independent variable: agreeableness | 0.394 | (0.343, 0.445) | 15.085^***^ | 0.100 | 135.163^***^ |
| Dependent variable: extra-family social relationship |  |  |  |  |  |
| Step 3: Extra-family social relationship predicts depressive symptoms |  |  |  |  |  |
| Independent variable: agreeableness | -0.526 | (-0.629, -0.423) | -9.999^***^ | 0.158 | 211.084^***^ |
| Mediator: extra-family social relationship | -0.113 | (-0.145, -0.080) | -6.820^***^ |  |  |
| Dependent variable: depressive symptoms |  |  |  |  |  |

Note: ^a^ Adjusted for all covariates.

^b^ Adjusted for all covariates except body mass index (BMI).

^***^ *P* < 0.001
